# Impact of regular physical activity on weekly warfarin dose requirement

**DOI:** 10.1007/s11239-015-1248-9

**Published:** 2015-08-04

**Authors:** Étienne Rouleau-Mailloux, Payman Shahabi, Stéphanie Dumas, Yassamin Feroz Zada, Sylvie Provost, Jason Hu, Jacqueline Nguyen, Nawal Bouchama, Ian Mongrain, Mario Talajic, Jean-Claude Tardif, Sylvie Perreault, Marie-Pierre Dubé

**Affiliations:** Beaulieu-Saucier Pharmacogenomics Centre, Montreal Heart Institute, Université de Montréal, Montreal, QC Canada; Department of Pharmacology, Faculty of Medicine, Université de Montréal, Montreal, QC Canada; Faculty of Pharmacy, Université of Montréal, Montreal, QC Canada; Faculty of Medicine, Université de Montréal, Montreal, QC Canada

**Keywords:** Warfarin, Dose, Physical activity, Interaction

## Abstract

Warfarin is an oral anticoagulant agent with a narrow therapeutic index. There is a marked inter- and intra-patient variability in warfarin dose requirement. All factors influencing warfarin response are not known and this study aims to evaluate if regular physical activity (RPA) is a determining factor. RPA level was collected with the Stanford Brief Activity Survey in 1064 incident warfarin users, as part of the Quebec Warfarin Cohort (QWC), and with the Global Physical Activity Questionnaire in 618 patients from the Montreal Heart Institute (MHI) Biobank. Linear regression was performed to model relationship of warfarin dose after 3 months of therapy in the QWC with RPA, while controlling for height, weight, age, *CYP2C9* (*2 and *3 alleles) and *VKORC1* (*2 allele) genotype. Warfarin dose of prevalent users was modeled in the MHI Biobank for replication. A higher level of physical activity was associated with higher doses of warfarin in both cohorts. In the QWC, physical activity could explain 5.4 % (*P* < 0.001) and 0.9 % (*P* = 3.23 × 10^−5^) of variance in dose, in univariate and multivariable models, respectively. Similarly, RPA was found to be associated with 1.7 % (*P* = 0.0012) and 0.5 % (*P* = 0.0391) of inter-individual variability in warfarin dose requirement before and after adjustment for other covariables, respectively. RPA is associated with higher warfarin dose requirement. The relevance of clinical recommendations on RPA to maintain a steady response to warfarin should be assessed in further studies.

**Electronic supplementary material:**

The online version of this article (doi:10.1007/s11239-015-1248-9) contains supplementary material, which is available to authorized users.

## Introduction

Warfarin is the most widely prescribed oral anticoagulant worldwide for the treatment and the prevention of thromboembolic disorders [1]. However, warfarin has a narrow therapeutic window. There is also a marked inter- and intra-individual variability in the dose requirement, so that the effective therapeutic dose may range from 1 to 20 mg per day [2]. The anticoagulation therapy with warfarin is monitored with regular measurement of the international normalized ratio (INR). Sub- and supra-therapeutic INR have been associated with an increased risk of thromboembolic and bleeding events, respectively [3, 4].

Several factors can influence the therapeutic warfarin dose. It has been estimated that height, weight and age predict 10–20 % of the variability in warfarin dose and concomitant medication is responsible for another 5–10 % [5]. Also, up to 40 % of warfarin dose variability is attributed to the genetic variants of two genes; *VKORC1,* the gene encoding the warfarin target, i.e. vitamin K epoxide reductase complex subunit 1 and; *CYP2C9*, the gene encoding the main warfarin metabolizing enzyme, i.e. cytochrome P-450 2C9. An estimated 30–40 % of the variation in patients’ response to warfarin remains to be determined [5]. Despite the great efforts made in recent years to provide a personalized warfarin therapy, up to 11 and 15 % of on-warfarin patients still suffer from hemorrhagic and thrombotic events, respectively, which can be ascribed to the unknown factors [6–9].

There exist very few studies that have investigated the potential impacts of regular physical activity (RPA) on the warfarin dose requirement and INR. A recent study showed that the RPA is associated with higher warfarin maintenance dose [10]; however, the results of this single study need to be confirmed by further research.

In the present study, we first investigated the association of RPA with warfarin dose in a large cohort of new users of warfarin. We then replicated our findings in an independent on-warfarin hospital-based population.

## Methods

### Discovery population

The association of RPA and warfarin dose requirement was first investigated in the Quebec Warfarin Cohort (QWC). The QWC is an observational, community-based, prospective, epidemiological cohort study of new warfarin users which aims to systematically identify the clinical, lifestyle and genetic determinants of response to warfarin. The patients were recruited consecutively between May 2010 and July 2013 at 18 anticoagulation clinics in the Quebec province of Canada, among which, the Montreal Heart Institute (MHI) was the leading and coordinating center. Patients were included in the QWC if they were aged more than 18 years; had at least one indication for warfarin therapy (except for deep venous thrombosis); and were expected to take warfarin for a treatment duration of 12 months or more. Also, the following patients were excluded from the study: patients who were prescribed warfarin for a deep vein thrombosis, pulmonary embolism or isolated left ventricular thrombosis; patients with at least one major bleeding episode, including gastro-intestinal bleeding and hemorrhagic stroke, within the past 3 months; and patients with cirrhosis, chronic hepatitis, icterus, end stage renal failure and mental illness. Following a short face-to-face recruitment interview, patients were followed-up for a period of 12 months (every 3 months) with 5 structured telephone questionnaires (Q); one baseline (Q0), three follow-up (Qi) and one final (Qf) questionnaire. Demographic data and information about indication for warfarin therapy were obtained at the recruitment interview and from medical charts. Medical history, cardiovascular history, life-style habits including smoking, diet and physical activity were captured by patient telephone interviews. RPA were obtained from Q0 and Qf.

The study was performed under the terms of the Declaration of Helsinki. Also, the study protocol was approved by the local review boards or ethics committees and all patients gave written informed consent.

**Fig. 1:**
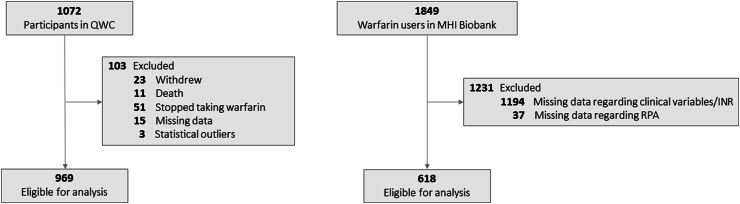
Flow diagram for patient inclusion into the QWC and the MHI Biobank study population

Of 1072 patients eligible for study, 23 patients withdrew from the study, 11 patients died and 51 patients stopped taking warfarin within the first three months of treatment. Moreover, 15 patients were excluded from the analysis due to missing data and 3 patients were categorized as outliers (Fig. 1). Accordingly, the test of association of RPA with warfarin dose at the 3-month time point was performed on the remaining 969 individuals. Warfarin dose was measured as the sum of patient-reported daily doses taken during the 7 days prior to the three-month patient interview. The concordance of patient self-reported warfarin doses with doses prescribed by physicians has been validated previously in a subset of 219 patients of the QWC [11].

In the QWC, the level of RPA was assessed by using the validated Stanford Brief Activity Survey (SBAS). This is a short checklist stratifying patients into one out of five statements that best describes the level of physical activity that a person currently performs when spending time on the job and separately during leisure time. A combined score provides classification into one of five intensity levels of RPA, of which the RPA level 1 and 5 represent the most sedentary and active patients, respectively [12]. Details concerning the SBAS scoring system are provided in the Supplementary Appendix.

### Replication cohort

The MHI Biobank is a hospital-based longitudinal observational study with over 18,000 patients who were recruited from different departments of the MHI for clinical and genetic research studies. Data about demographics, lifestyle (including physical activity) and personal and family medical history were captured at a baseline interview using a 35-page questionnaire and validated using hospital medical records. The patients are followed-up every 4 years using a complete follow-up questionnaire. The MHI Biobank is also automatically updated weekly from the hospital’s electronic health record of the patients. The MHI ethics committee approves the project and informed consent is obtained from all participants.

There was no overlap of patients between the QWC and the MHI Biobank. We identified 1849 patients who reported using warfarin at the time of recruitment into the MHI Biobank. Warfarin dosage was obtained directly from the MHI anticoagulation clinic records and corresponds to the closest recoded dose to the date of the baseline visit for the MHI Biobank study. Participants were excluded if the time between the recruitment and the available warfarin’s dosage was more than 12 months. All selected participants had been taking warfarin for more than 3 months, as confirmed from INR laboratory measures from patients’ medical charts. Data for RPA, age, sex, weight, height, ethnicity and genetic information was taken directly from the MHI Biobank baseline questionnaire. After the data quality processing (Fig. 1), 618 warfarin users were available for the final analysis from the MHI Biobank population.

RPA was assessed using the second version of the Global Physical Activity Questionnaire (GPAQ). The GPAQ is a validated questionnaire developed by the World Health Organization (WHO) and comprising 16 questions on the level of physical activity in three settings, i.e. work, travel and recreational activities, and also on sedentary behaviour [13–15]. The patients were classified into three groups based on the intensity level of RPA; the RPA level 1 represented the most sedentary and the RPA level 3 the most active patients. Details about the GPAQ are provided in the Supplementary Appendix.

### Genotyping

Genotyping was performed at the Beaulieu-Saucier Pharmacogenomics Centre of the MHI. For the QWC, genotyping of *CYP2C9*, and *VKORC1* was performed using iPLEX^®^ ADME PGx Panel (Sequenom, Inc.). For the MHI Biobank, genotyping was performed with the Illumina Exome BeadChip from which the data on CYP2C9*2 (430C>T; rs1799853) and CYP2C9*3 (1075A>C; rs1057910) was available for the analysis but VKORC1*2 (−1639G>A; rs9923231) was missing. Given that VKORC1*2 is located in the promoter region of *VKORC1* and that an Exome chip primarily genotypes the protein-coding parts of the genome, we were not able to search for a genotyped SNP in the region with a strong linkage disequilibrium with the *VKORC1* SNP and, thereby, we were unable to impute with confidence the missing SNP in the MHI Biobank samples. More details about genotyping are found in the Supplementary Appendix.

### Statistical analysis

Statistical analyses were performed with SAS 9.3 (SAS Institute Inc., Cary, NC). Warfarin dosage was transformed using a natural logarithm to ensure normality distribution of the values. The level of RPA reported at baseline and the patient-reported warfarin dose at the three-month interview was used in the QWC. For the MHI Biobank, the RPA reported at baseline and the warfarin dose reported in the medical record that was the closest to the baseline date was used.

For both patient populations, univariate modeling was first performed with relevant and available variables: age, sex, weight, height, level of RPA, *CYP2C9* and *VKORC1* polymorphisms (*VKORC1* genetic variant was not available for the MHI Biobank), target INR and indication of warfarin therapy. Variable selection for multiple regression modeling was based on multivariable stepwise linear regression with those variables (inclusion at *P* < 0.05). Multiple regression with a general linear model (GLM) for warfarin dose included RPA and selected covariates. Genotypes were coded as 0, 1 and 2 according to the number of alternative (minor) alleles.

## Results

The baseline characteristics of patients enrolled in the QWC (n = 969) and in the MHI Biobank (n = 618) are shown in Table 1. The main indication for warfarin therapy in the QWC was paroxysmal and chronic atrial fibrillation, accounting for a total of 719 (74.20 %) patients. In the QWC and the MHI Biobank, the mean warfarin dose requirement was 33.15 mg/week (range 2.25−126; SD = 15.48) and 34.37 mg/week (range 5.53–95.00; SD = 14.70), respectively. In addition, in the both populations, the recruited patients were primarily of Caucasian descent (99.8 and 99.7 % for the QWC and the MHI Biobank, respectively).

**Table 1:** Demographic characteristics of the QWC and the MHI Biobank study populations

Variable	QWC (n = 969)	MHI Biobank (n = 618)
Median age (years) (range)	71 (18–95)	69 (27–90)
Male sex, no. (%)	592 (61.10)	402 (64.63)
Race or ethnic group^a^, no. (%)		
White	938 (96.80)	620 (99.68)
Black	10 (1.03)	0 (0)
Asian	4 (0.41)	0 (0)
Other	17 (1.75)	2 (0.32)
Mean weight (kg) (range)	81.02 (36.5–215.9)	80.72 (46.0–154.0)
Mean height (m) (range)	1.68 (1.22–1.96)	1.68 (1.38–1.94)
Primary warfarin indication, no. (%)		
Mitral stenosis	11 (1.13)	N/A
Paroxysmal atrial fibrillation	394 (40.66)	N/A
Chronic atrial fibrillation	325 (33.54)	N/A
Atrial flutter	89 (9.18)	N/A
Mitral valve replacement	49 (5.06)	N/A
Aortic valve replacement	93 (9.60)	N/A
Other	8 (0.82)	N/A
Stable warfarin dose		
Mean (mg/week ± SD)	33.15 ± 15.48	34.37 ± 14.7
Range (mg/week)	2.25–126.00	5.53–95.00
Target INR, no. (%)		
2.0–3.0	854 (88.13)	437 (70.48)
2.5–3.5	115 (11.87)	183 (29.52)
CYP2C9*2 (rs1799853), no. (%)		
No variants	714 (73.68)	491 (79.07)
Heterozygous	236 (24.35)	130 (20.93)
Homozygous	19 (1.96)	0 (0)
CYP2C9*3 (rs1057910), no. (%)		
No variants	841 (86.80)	531 (85.37)
Heterozygous	124 (13.00)	87 (13.99)
Homozygous	4 (0.41)	4 (0.64)
VKORC1*2 (rs9923231), no. (%)		
No variants	364 (37.56)	N/A
Heterozygous	449 (46.34)	N/A
Homozygous	156 (16.10)	N/A

*SD* standard deviation

^a^Race or ethnic group was self-reported

### RPA and warfarin dose in the QWC

**Fig. 2:**
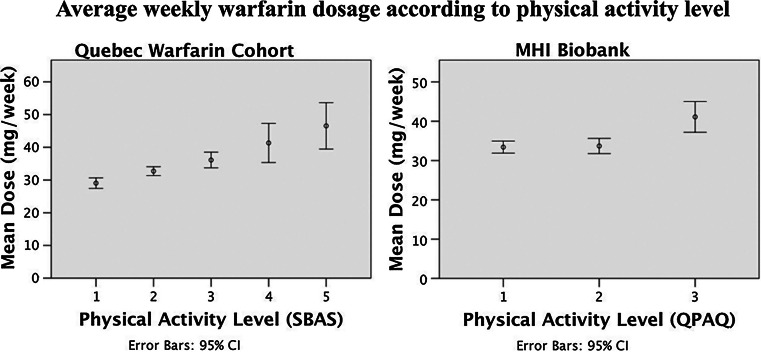
Univariate regression analyses of average weekly warfarin dose requirement in function of regular physical activity in the QWC and the MHI Biobank

In the QWC, the level of RPA at baseline was significantly and positively (*P* < 0.001, R^2^ = 5.4 %) associated with the required dose of warfarin per week at 3 month (Fig. 2), so that the lowest dose of warfarin (29.05 ± 13.27 mg) was required for the patients of the most sedentary group (RPA level 1; n = 260), followed by those in the RPA level 2 (n = 461; 32.61 ± 14.93 mg), RPA level 3 (n = 179; 36.26 ± 16.27 mg), RPA level 4 (n = 37; 41.43 ± 17.92 mg) and the RPA level 5 (n = 32; 46.56 ± 19.69 mg), i.e. the most active group.

Multivariable stepwise linear regression for the modeling of log transformed weekly warfarin dose at month 3 was used to select clinical variables including age (*β* = −0.012, *P* < *0*.001), height (*β* = 0.510, *P* = 0.002) and weight (*β* = 0.002, *P* = 0.007), but not gender (*β* = −1.476, *P* = 0.140), as independent predictors. The GLM including *CYP2C9* and *VKORC1* genotypes, age, height, weight and target INR, but excluding the level of RPA, explained 50.8 % of the variability of the observed warfarin dose (*P*_*model*_ = 2.06 × 10^−143^). The same model including the level of RPA produced the best model for estimating warfarin dose at 3 months in this population (R^2^ = 51.7 %, *P*_*model*_ = 4.73 × 10^−146^) (Tables 2, 3). In fact, the addition of RPA to the multiple regression model including clinical and genetic predictors resulted in a gain of 0.9 % in the model’s prediction of weekly warfarin dose requirement. In this model, RPA was independently associated with warfarin dose (*P* = 3.23 × 10^−5^) (Table 3).

**Table 2:** Regression equations for the modeling of weekly warfarin dose in the QWC according to genetic and clinical predictor variables and RPA

Variable (s)	Regression equation	*P*	R^2^ for model (%)
RPA	Ln (dose) = 3.148 + 0.118 (RPA)	<0.001	5.4
Target INR	Ln (dose) = 2.037 + 0.531 (target INR)	<0.001	3.3
Genetic variables	Ln (dose) = 3.761 − 0.194 (CYP2C9*2) − 0.435 (CYP2C9*3) − 0.322 (VKORC1*2)	<0.001	36.0
Clinical variables^a^	Ln (dose) = 3.193 − 0.012 (age) + 0.510 (height) + 0.002 (weight)	<0.001	15.4
Genetic and clinical variables + target INR	Ln (dose) = 2.812 − 0.211 (CYP2C9*2) − 0.428 (CYP2C9*3) − 0.316 (VKORC1*2) + 0.148 (target INR) − 0.010 (age) + 0.594 (height) + 0.003 (weight)	<0.001	50.8
Genetic and clinical variables + target INR + RPA	Ln (dose) = 2.735 + 0.053 (RPA) − 0.215 (CYP2C9*2) − 0.427 (CYP2C9*3) − 0.315 (VKORC1*2) + 0.129 (target INR) − 0.008 (age) + 0.518 (height) + 0.003 (weight)	<0.001	51.7

RPA coded as 1, 2, 3, 4, or 5 according to SBAS level; Genetic variables: CYP2C9*2, CYP2C9*3 and VKORC1*2 were coded as 0, 1, or 2 according to the number of variant (minor) alleles; Age in years; Height in meters; Weight in kilograms

^a^Age, height and weight

**Table 3:** Factors included in the GLM of weekly warfarin dose in the QWC

Parameter	Estimate	SE	*P*
RPA	0.0525	0.0126	3.23 × 10^−5^
CYP2C9*2	−0.2146	0.0220	1.61 × 10^−21^
CYP2C9*3	−0.4269	0.0305	1.02 × 10^−40^
VKORC1*2	−0.3149	0.0153	5.70 × 10^−78^
Target INR	0.1292	0.0738	0.0801
Age	−0.0081	0.0011	1.95 × 10^−12^
Height	0.5184	0.1238	3.08 × 10^−5^
Weight	0.0034	0.0007	2.25 × 10^−7^

969 patients were included in the analysis; *P* value for the model = 4.73 × 10^−146^ and R^2^ for the model = 0.5171

*SE* standard error

### RPA and warfarin dose in MHI Biobank

In the MHI Biobank, in univariate analysis, the level of RPA was significantly associated with the warfarin dose in univariate analysis (*P* = 0.0012; R^2^ = 1.7 %), so that the lowest dose of warfarin (33.46 ± 14.56 mg) was required for the most sedentary group (RPA level 1; n = 351), followed by those in the RPA level 2 (n = 206; 33.74 ± 14.00 mg) and the RPA level 3 (n = 64; 41.09 ± 15.68 mg), the most active group for the GPAQ questionnaire (Fig. 2). Comparable to what was observed in the QWC, age (*β* = −0.165, *P* < *0*.001), height (*β* = 0.203, *P* < *0*.001) and weight (*β* = 0.100, *P* = 0.004), but not gender (*β* = −0.009, *P* = 0.962), were found in multivariable regression analysis to be independent predictors of warfarin dose in the MHI Biobank. In this population, the GLM including *CYP2C9* polymorphisms, age, height, weight and target INR, but excluding the level of RPA, explained 33.8 % of the variability of the observed warfarin dose (*P*_*model*_ < 0.0001). The same model including the level of RPA explained 34.31 % of the variability in the weekly warfarin dose requirement (*P*_*model*_ < 0.0001), i.e. 0.5 % more than that obtained by the model without RPA. Moreover, in the final model, RPA was independently associated with warfarin dose (*P* = 0.0391).

## Discussion

Physical activity has long been recognized as a non-pharmacological strategy to achieve a healthy body weight, improve mood, reduce stress and prevent a number of chronic diseases including cardiovascular disease, diabetes and cancer. There is a growing body of evidence suggesting that beyond the numerous and well-known beneficial physiological effects of physical activity, it may have significant effects on drug pharmacokinetics and response. [1, 16–19]. The evaluation of drug-exercise interaction is of particular importance for drugs with a narrow therapeutic index, such as warfarin, for which modest changes in the pharmacokinetic parameters may have a great impact on the drug’s safety and toxicity. Nonetheless, the pharmacokinetic parameters of most medications are typically assessed under resting and non-stressful conditions and consequently, the potential for drug-exercise interactions may have been under reported.

In the present study, we demonstrated that there is a significantly positive relationship between the level of RPA and warfarin dose requirement, such that the more active patients require higher doses of warfarin. This association was maintained in two different study populations. First, in the QWC consisting of incident warfarin users and tested using warfarin dose requirement after 3 months of therapy, and then replicated in the MHI Biobank study using prevalent warfarin users with an unselected measure of warfarin dose. In the QWC, based on univariate regression analysis, physical activity was associated with 5.4 % of variance in stable warfarin dose. Considering other covariables, RPA could explain 0.9 % of inter-individual variation in dose requirement. Likewise, in the MHI Biobank, in univariate analysis, RPA was found to be associated with 1.7 % of variability in the weekly warfarin dose requirement and after adjustment for other predictors, RPA explained 0.5 % of differences in warfarin dose. Two different RPA questionnaires were used for this study, which further adds to the validity of the association.

Our findings are consistent with and extend those of Shendre et al. [10], who recently showed that compared with inactive patients, those with RPA, defined as having exercise ≥30 min ≥3 times/week, needed a 6.9 % higher dose of warfarin. Despite the different methods of physical activity assessment used, both studies revealed similar findings regarding the association of physical activity with warfarin dose requirement, which could be interpreted as an additional evidence for the robustness of this association.

In their paper, Shendre et al. also demonstrated that RPA is associated with a 38 % lower risk of major hemorrhage in an inception cohort of warfarin new-users [10], suggesting that physical activity may have additional protective effects in on-warfarin patients, that is beyond its beneficial physiological actions. In our study, we did not test for the association of physical activity and the risk of bleeding events since data were not available at the time of writing. Our results also support those of Shibata et al. who originally reported that increased physical activity is inversely associated with decreased INR in three warfarin stabilized patients [20].

The warfarin pharmacokinetic interaction with physical activity could be attributable to different mechanisms. Warfarin has a low hepatic extraction ratio and the effectiveness of such drugs is greatly influenced by their unbound fraction in the blood (distribution) and the level of drug-metabolizing enzyme activity in the liver (metabolism) [1, 21]. Indeed, of the total amounts of warfarin in the blood, 99 % is bound to plasma proteins, i.e. mainly albumin. Exercise can reduce the level of free (unbound) warfarin through inducing the synthesis of plasma proteins that have generally a high affinity for the drug [18, 22–24]. Accordingly, a greater dose of warfarin would have to be administered to reach comparable plasma concentration in the more active patients. Furthermore, RPA may induce the expression and activity of hepatic microsomal enzymes involved in the metabolism of warfarin [18]. For instance, Chien et al. recently showed that an 8-week exercise program is associated with a significant increase in the liver microsomal CYP2C9 activity in rats [25]. These findings and their clinical significance will need to be corroborated with further human studies. It should be noted that during physical activity, blood flow is shunted away from the liver to the working muscles, resulting in a major change in the metabolism of the drugs with a high hepatic extraction. But counter to that, the hepatic clearance of the medications with a low hepatic extraction, such as warfarin, is nearly completely independent to the liver blood flow. Therefore, the exercise-induced blood redistribution may not exert a notable effect on warfarin pharmacokinetics. Other than changes in the blood flow, the mechanisms by which physical activity can acutely modulate the efficacy of warfarin have not been investigated so far.

In this study, physical activity assessment was based on indirect and self-reported subjective measures. More direct measures of exercise that can more objectively assess energy expenditure or physical movement are generally more accurate than the indirect methods. Such method of physical activity capture would also have been better suited to detect intra-patient variability in physical activity in relation to changes in warfarin dose requirements. However, direct measure has several drawbacks which may limit their use in the clinical and research settings. Indeed, direct measurement devices are not able to capture swimming and biking; need to be worn generally for a long time; are expensive, expert-dependent and time-consuming; and may not be readily accessible for regular use.

In addition to the genetic factors, certain clinical factors like dietary habits, including alcohol consumption and vitamin K intake (diet rich in green vegetables) and concomitant medications (such as amiodarone) contribute to the capricious nature of response to warfarin [26]. This study did not investigate the possible correlation between physical activity and these factors which is acknowledged as a limitation of the study. Furthermore, we recognize that the study participants are primarily Caucasians, potentially restricting the applicability of our findings in other ancestries like African-Americans, Asians and Hispanics. Further studies are required to elucidate the influences of physical activity in these populations.

Of note, the MHI Biobank cohort was not ideally suited for this study as there was a variable time lapse between RPA questionnaire capture at cohort baseline, and the collection of warfarin dosage data from the hospital charts which varied between patients. This could have contributed to some loss in power in the association signal.

Overall, our results support the notion that there is an interaction between RPA and the required dose of warfarin. These findings do not warrant a recommendation for routine use of an exercise questionnaire in daily clinical practice. Likewise, considering the sustained beneficial effects of physical activity on health, recommendations with regards to physical activity should not be revoked for warfarin-treated patients. Nonetheless, the convergent evidence from the present and past studies does call for some level of awareness by care providers of the potential impact of physical activity on warfarin dosing. To our best knowledge, the only recommendation found in International Guidelines for treatment with warfarin pertaining to physical activity states that patients should avoid activities which may results in serious fall and injuries. Guidelines do not yet provide advice on maintaining a regular level of physical activity. Further well-designed, large-sample studies are needed to provide sufficient level of evidence toward changes in Guideline recommendations. Ours is the second study in this direction. The exercise-drug interaction could also be of importance for other medications with a narrow therapeutic index. For instance, it is well known that exercise can also reduce renal blood and modify therapies that are primarily eliminated by the kidneys, such as for novel oral anticoagulants [16]. Also, the irregular practice of physical activities may be expected to induce instability in response to the drugs with a narrow therapeutic window. These hypotheses and the clinical implications of the results of the present work should be investigated in further studies.

## Electronic supplementary material

Supplementary material 1 (DOCX 43 kb)

## References

[1] Lenz TL, Lenz NJ, Faulkner MA (2004). Potential interactions between exercise and drug therapy. Sports Med.

[2] Johnson JA, Gong L, Whirl-Carrillo M (2011). Clinical Pharmacogenetics Implementation Consortium Guidelines for CYP2C9 and VKORC1 genotypes and warfarin dosing. Clin Pharmacol Ther.

[3] Wallvik J, Sjalander A, Johansson L (2007). Bleeding complications during warfarin treatment in primary healthcare centres compared with anticoagulation clinics. Scand J Prim Health Care.

[4] Newman DH, Zhitomirsky I (2006) The prevalence of nontherapeutic and dangerous international normalized ratios among patients receiving warfarin in the emergency department. Ann Emerg Med 48:182–189, 189 e1 10.1016/j.annemergmed.2005.12.010 16953531

[5] Jonas DE, McLeod HL (2009). Genetic and clinical factors relating to warfarin dosing. Trends Pharmacol Sci.

[6] Aithal GP, Day CP, Kesteven PJ, Daly AK (1999). Association of polymorphisms in the cytochrome P450 CYP2C9 with warfarin dose requirement and risk of bleeding complications. Lancet.

[7] Fanikos J, Grasso-Correnti N, Shah R (2005). Major bleeding complications in a specialized anticoagulation service. Am J Cardiol.

[8] Samsa GP, Matchar DB (2000). Relationship between test frequency and outcomes of anticoagulation: a literature review and commentary with implications for the design of randomized trials of patient self-management. J Thromb Thrombolysis.

[9] Taube J, Halsall D, Baglin T (2000). Influence of cytochrome P-450 CYP2C9 polymorphisms on warfarin sensitivity and risk of over-anticoagulation in patients on long-term treatment. Blood.

[10] Shendre A, Beasley TM, Brown TM (2014). Influence of regular physical activity on warfarin dose and risk of hemorrhagic complications. Pharmacotherapy.

[11] Dumas S, Rouleau-Mailloux E, Barhdadi A (2014). Validation of patient-reported warfarin dose in a prospective incident cohort study. Pharmacoepidemiol Drug Saf.

[12] Taylor-Piliae RE, Norton LC, Haskell WL (2006). Validation of a new brief physical activity survey among men and women aged 60-69 years. Am J Epidemiol.

[13] Bull FC, Maslin TS, Armstrong T (2009). Global physical activity questionnaire (GPAQ): nine country reliability and validity study. J Phys Act Health.

[14] Herrmann SD, Heumann KJ, Der Ananian CA, Ainsworth BE (2013). Validity and Reliability of the Global Physical Activity Questionnaire (GPAQ). Meas Phys Educ Exerc Sci.

[15] Hoos T, Espinoza N, Marshall S, Arredondo EM (2012). Validity of the Global Physical Activity Questionnaire (GPAQ) in adult Latinas. J Phys Act Health.

[16] Khazaeinia T, Ramsey AA, Tam YK (2000). The effects of exercise on the pharmacokinetics of drugs. J Pharm Pharm Sci.

[17] Lenz TL (2011). The effects of high physical activity on pharmacokinetic drug interactions. Expert Opin Drug Metab Toxicol.

[18] Persky AM, Eddington ND, Derendorf H (2003). A review of the effects of chronic exercise and physical fitness level on resting pharmacokinetics. Int J Clin Pharmacol Ther.

[19] van Baak MA (1990). Influence of exercise on the pharmacokinetics of drugs. Clin Pharmacokinet.

[20] Shibata Y, Hashimoto H, Kurata C (1998). Influence of physical activity on warfarin therapy. Thromb Haemost.

[21] Dossing M (1985). Effect of acute and chronic exercise on hepatic drug metabolism. Clin Pharmacokinet.

[22] Larsen FG, Larsen CG, Andersen S (1986). Warfarin binding to plasma albumin, measured in patients and related to fatty acid concentrations. Eur J Clin Invest.

[23] Spector AA, Santos EC, Ashbrook JD, Fletcher JE (1973). Influence of free fatty acid concentration on drug binding to plasma albumin. Ann N Y Acad Sci.

[24] Vorum H, Honore B (1996). Influence of fatty acids on the binding of warfarin and phenprocoumon to human serum albumin with relation to anticoagulant therapy. J Pharm Pharmacol.

[25] Chien KJ, Horng CT, Chao HR (2013). The influence of running exercise training on pharmacokinetics of meloxicam in rats. Life Sci J.

[26] Jonas DE, McLeod HL (2009). Genetic and clinical factors relating to warfarin dosing. Trends Pharmacol Sci.

