# Supplementary material for: Impact of regular physical activity on weekly warfarin dose requirement
Source: J Thromb Thrombolysis. 2015 Aug 4;41:328–35. doi: 10.1007/s11239-015-1248-9 (PMC4735233; doi:10.1007/s11239-015-1248-9)
Supplement: Supplementary file 1 — Supplementary material 1 (DOCX 43 kb) [file 11239_2015_1248_MOESM1_ESM.docx]

Supplementary Appendix

Impact of Regular Physical Activity on Weekly Warfarin Dose Requirement

Étienne Rouleau-Mailloux^A,B^, Payman Shahabi^A,D^, Stéphanie Dumas^A,C^, Yassamin Feroz Zada^A^, Sylvie Provost^A^, Jason Hu^A^, Jacqueline Nguyen^A^, Nawal Bouchama^A^, Ian Mongrain^A^, Mario Talajic^A,D^, Jean-Claude Tardif^A,D^, Sylvie Perreault^C,D^, Marie-Pierre Dubé^A,D^**^*^**

^A^ Beaulieu-Saucier Pharmacogenomics Centre, Montreal Heart Institute, Université de Montréal, Montreal, Quebec, Canada

^B^ Department of Pharmacology, Faculty of Medicine, Université de Montréal, Montreal, Quebec, Canada

^C^ Faculty of Pharmacy, Université of Montréal, Montreal, Quebec, Canada

^D^ Faculty of Medicine, Université de Montréal, Montreal, Quebec, Canada

***Corresponding author:**

Marie-Pierre Dubé, PhD

Beaulieu-Saucier Université de Montréal Pharmacogenomics Center

5000 Bélanger Street

Montreal H1T 1C8, QC, Canada

Phone: (514) 670-7670

Fax: (514) 670-7671

Email: marie-pierre.dube@umontreal.ca

**Stanford Brief Activity Survey (SBAS)**

The SBAS is a self-administered questionnaire that provides a quick assessment of the amount and intensity of physical activity (PA) that a person typically performs on the job and during leisure time, on the basis of a single statement for each [1,2].

The patients are asked to read throughout the entire questionnaire and to select the answer that best describes his/her activity within the past year. The SBAS has two parts. The first part represents five different patterns on on-the-job activity, ranging from mostly sedentary to hard physical labor (Table S1). The second part represents five patterns of leisure-time activity, ranging from sedentary to regular vigorous activity on five or more days per week (Table S2). The respondent is asked to choose one pattern that best represents his/her on-the-job activity and one pattern that best represents his/her leisure-time activity pattern [1,2].

Each respondent is categorized into one out of five SBAS categories (inactive, light, moderate, hard and very-hard intensities) using a color-coded scoring table (Figure S1). On-the-job activity patterns are found on the vertical axis of the table (response options A–E; i.e. sedentary—hard physical labor), while leisure-time activity patterns are found on the horizontal axis of the table (response options F–J; i.e. sedentary—regular vigorous activity). The intersection of these 2 responses on the color-coded scoring table, determines the respondent’s current activity pattern. For instance, a respondent who works in an office and sits most of the day at a desk and rides a bicycle for 30 minutes/day every day the week, should select ‘‘B’’ as his/her on the-job activity and ‘‘J’’ as his/her leisure-time activity. The intersection of these two responses on the color-coded table would place them in the very hard-intensity activity category (horizontal lines) [1,2].

**Table S1.** Stanford Brief Activity Survey of on-the-job activity

| **A. □** | If you have no job or regular work, check box A and go on to Table S2. |
| --- | --- |
| **B. □** | I spent most of the day sitting or standing. When I was at work, I did such things as writing, typing, talking on the telephone, assembling small parts, or operating a machine that takes very little exertion or strength. If I drove a car or truck while at work, I did not lift or carry anything for more than a few minutes each day. |
| **C. □** | I spent most of the day walking or using my hands and arms in work that required moderate exertion. When I was at work, I did such things as delivering mail, patrolling on guard duty, doing mechanical work on automobiles or other large machines, house painting, or operating a machine that requires some moderate-activity work of me. If I drove a truck or lift, my job required me to lift and carry things frequently. |
| **D. □** | I spent most of the day lifting or carrying heavy objects or moving most of my body in some other way. When I was at work, I did such things as stacking cargo or inventory, handling parts or materials, or doing work like that of a carpenter who builds structures or a gardener who does most of the work without machines. |
| **E. □** | I spent most of the day doing hard physical labor. When I was at work, I did such things as digging or chopping with heavy tools or carrying heavy loads (bricks, for example) to the place where they were to be used. If I drove a truck or operated equipment, my job also required me to do hard physical work most of the day with only short breaks. |

**Table S2.** Stanford Brief Activity Survey of leisure-time activity

| **F. □** | Most of my leisure time was spent without very much physical activity. I mostly did things like watching television, reading, or playing cards. If I did anything else, it was likely to be light chores around the house or yard or some easy-going game like bowling or catch. Only occasionally, no more than once or twice a month, did I do anything more vigorous, like jogging, playing tennis, or active gardening. |
| --- | --- |
| **G. □** | Weekdays, when I got home from work, I did few active things, but most weekends I was able to get outdoors for some light exercise—going for walks, playing a round of golf (without motorized carts), or doing some active chores around the house. |
| **H. □** | Three times per week, on average, I engaged in some moderate activity, such as brisk walking or slow jogging, swimming, or riding a bike for 15–20 minutes or more, or I spent 45 minutes to an hour or more doing moderately difficult chores, such as raking or washing windows, mowing the lawn or vacuuming, or playing games such a doubles tennis or basketball. |
| **I. □** | During my leisure time over the past year, I engaged in a regular program of physical fitness involving some kind of heavy physical activity at least three times per week. Examples of heavy physical activity are jogging, running, or riding fast on a bicycle for 30 minutes or more; heavy gardening or other chores for an hour or more; active games or sports such as handball or tennis for an hour or more; or a regular program involving calisthenics and jogging or the equivalent for 30 minute or more. |
| **J. □** | Over the past year, I engaged in a regular program of physical fitness along the lines described in the last paragraph (I), but I did it almost daily—five or more times per week. |

**Figure S1.** Stanford Brief Activity Survey color-coded scoring table

|  | Leisure-time Activity (F-J) | | | | | |
| --- | --- | --- | --- | --- | --- | --- |
| On-the-job Activity (A-E) |  | F | G | H | I | J |
|  | A |  |  |  |  |  |
|  | B |  |  |  |  |  |
|  | C |  |  |  |  |  |
|  | D |  |  |  |  |  |
|  | E |  |  |  |  |  |

Activity categories are represented by different patterns as follows: inactive = vertical lines; light-intensity activity = trellis pattern; moderate-intensity activity = solid white; hard-intensity activity = solid black, and very hard-intensity activity = horizontal lines. Refer to Table S1 and S2 for explanations of A–E and F–J, respectively.

**Global Physical Activity Questionnaire (GPAQ)**

The GPAQ was developed in 2002 by the World Health Organization (WHO) as part of the WHO STEPwise Approach to Chronic Disease Risk Factor Surveillance for PA observation. Since 2004, the WHO has recommended that it be used in the surveillance of PA [3].

The GPAQ consists of 16 questions (Table S3) and designed to cover several components of PA, such as intensity, duration and frequency and to assess an individual’s level of PA in three domains: at work (which includes paid and unpaid work, in and outside of the home), for transport (to get to and from places), and during leisure time [3-5]. The rationale for asking the questions from all three domains is that some people are physically active in all three domains while others may not be active in any of the settings [3].

According to the GPAQ, each person is categorized into one of the three levels of PA, i.e. high, moderate and low, based on the total time he/she has spent in PA during a typical week and the intensity of the activity performed (Table S4) [3,6]. The intensity of PA is determined using metabolic equivalent (MET) that is the ratio of a person's working metabolic rate relative to the resting metabolic rate. One MET is defined as the energy cost of sitting quietly, and is equivalent to a caloric consumption of 1 kcal/kg/hour [3]. When determining a person’s GPAQ category, MET values are applied to the time variables according to the intensity (moderate or vigorous) of the activity such that 4 METs get assigned to the time spent in moderate activities, cycling and walking, and 8 METs to the time spent in vigorous activities [3].

It is noteworthy to mention that several studies have tested the validity and reliability of the GPAQ. The results indicate that the GPAQ is valid and reliable, but also adaptable to incorporate cultural and other differences [4-9].

**Table S3.** Global Physical Activity Questionnaire

| **Questions** | | **Answer** | **Code** |
| --- | --- | --- | --- |
| Activity at work | | | |
| 1 | Does your work involve vigorous-intensity activity that causes large increases in breathing or heart rate like *[carrying or lifting* *heavy loads, digging or construction work*] for at least 10 minutes continuously?  *[INSERT EXAMPLES] (USE SHOWCARD)* | Yes 1  No 2  If No, go to P4 | P1 |
| 2 | In a typical week, on how many days do you do vigorous intensity activities as part of your work? | Number of days └─┘ | P2 |
| 3 | How much time do you spend doing vigorous-intensity activities at work on a typical day? | Hours : minutes └─┴─┘: └─┴─┘  hrs mins | P3  (a-b) |
| 4 | Does your work involve moderate-intensity activity that causes small increases in breathing or heart rate such as brisk walking *[or carrying light loads*] for at least 10 minutes continuously?  *[INSERT EXAMPLES] (USE SHOWCARD)* | Yes 1  No 2  If No, go to P7 | P4 |
| 5 | In a typical week, on how many days do you do moderate-intensity activities as part of your work? | Number of days └─┘ | P5 |
| 6 | How much time do you spend doing moderate-intensity activities at work on a typical day? | Hours : minutes └─┴─┘: └─┴─┘  hrs mins | P6  (a-b) |
| Travel to and from places | | | |
| 7 | Do you walk or use a bicycle (*pedal cycle*) for at least 10 minutes continuously to get to and from places? | Yes 1  No 2  If No, go to P10 | P7 |
| 8 | In a typical week, on how many days do you walk or bicycle for at least 10 minutes continuously to get to and from places? | Number of days └─┘ | P8 |
| 9 | How much time do you spend walking or bicycling for travel on a typical day? | Hours : minutes └─┴─┘: └─┴─┘  hrs mins | P9  (a-b) |
| Recreational activities | | | |
| 10 | Do you do any vigorous-intensity sports, fitness or recreational (*leisure*) activities that cause large increases in breathing or heart rate like [*running or football,]* for at least 10 minutes continuously?  *[INSERT EXAMPLES] (USE SHOWCARD)* | Yes 1  No 2  If No, go to P13 | P10 |
| 11 | In a typical week, on how many days do you do vigorous-intensity sports, fitness or recreational (*leisure*) activities? | Number of days └─┘ | P11 |
| 12 | How much time do you spend doing vigorous-intensity sports, fitness or recreational activities on a typical day? | Hours : minutes └─┴─┘: └─┴─┘  hrs mins | P12  (a-b) |
| 13 | Do you do any moderate-intensity sports, fitness or recreational *(leisure*) activities that causes a small increase in breathing or heart rate such as brisk walking*,*(*cycling, swimming, volleyball*)for at least 10 minutes continuously?  *[INSERT EXAMPLES] (USE SHOWCARD)* | Yes 1  No 2  If No, go to P16 | P13 |
| 14 | In a typical week, on how many days do you do moderate-intensity sports, fitness or recreational (*leisure*) activities? | Number of days └─┘ | P14 |
| 15 | How much time do you spend doing moderate-intensity sports, fitness or recreational (*leisure*) activities on a typical day? | Hours : minutes └─┴─┘: └─┴─┘  hrs mins | P15  (a-b) |
| Sedentary behavior | | | |
| 16 | How much time do you usually spend sitting or reclining on a typical day? | Hours : minutes └─┴─┘: └─┴─┘  hrs mins | P16  (a-b) |

**Table S4.** Physical activity levels according to Global Physical Activity Questionnaire

| **Category** | **Criteria** |
| --- | --- |
| **High** | Vigorous-intensity activity on ≥3/week and accumulating at least 1500 MET-minute/week *OR* |
|  | ≥7 days of any combination of walking, moderate-intensity or vigorous-intensity activity achieving ≥3000 MET-minute/week |
| **Moderate** | ≥3 days/week of vigorous-intensity activity totaling 60 minutes *OR* |
|  | ≥5 days/week of moderate-intensity activity of ≥150 minutes *OR* |
|  | ≥5 days/week of any combination of walking, moderate-intensity or vigorous-intensity activities achieving ≥600 MET-minutes/week |
| **Low** | Does not meet the criteria for moderate or high |

**Genotyping**

DNA was extracted from 1 ml of whole blood using the QiaSymphony DNA midi kit version 1.1 (Qiagen, Garstilgweg, Switzerland) and quantified using the QuantiFluor™ dsDNA System (Promega, Madison, WI).

In the Quebec Warfarin Cohort (QWC), genotyping was performed using iPLEX® ADME PGx Panel (Sequenom, Inc.) and data on *CYP2C9*2, *3* and *VKORC1*2* were retrieved. The platform contains a set of 192 SNPs in 36 pharmacogenetically relevant genes including *ABCB1, CYP2E1, SLC22A2, ABCC2, CYP3A4, SLC22A6, ABCG2, CYP3A5, SLCO1B1, COMT, DPYD, SLCO1B3, CYP1A1, GSTM1, SLCO2B1, CYP1A2, GSTP1, SULT1A1, CYP2A6, GSTT1, TPMT, CYP2B6, GSTT2, UGT1A1, CYP2C19, NAT1, UGT2B15, CYP2C8, NAT2, UGT2B17, CYP2C9, SLC15A2, UGT2B7, CYP2D6, SLC22A1* and *VKORC1* (https://www.sequenom.com/). The call rate for the three studied SNPs was 100% in the QWC.

In the Montreal Heart Institute (MHI) Biobank, genotyping was performed using the Illumina Exome BeadChip and data on *CYP2C9*2* and **3* were retrieved. The platform contains >240,000 markers, including 219,621 nonsynonymous SNPs, from 12,000 individual exome and whole-genome sequences (http://www.illumina.com/). The call rate for the two studied SNPs was 100% in the MHI Biobank.

**References:**

1. Taylor-Piliae RE, Norton LC, Haskell WL, et al. [Validation of a new brief physical activity survey among men and women aged 60-69 years.](http://www.ncbi.nlm.nih.gov/pubmed/16840522) Am J Epidemiol 2006;164:598-606.
2. [Taylor-Piliae RE](http://www.ncbi.nlm.nih.gov/pubmed/?term=Taylor-Piliae%20RE%5BAuthor%5D&cauthor=true&cauthor_uid=20231759), [Fair JM](http://www.ncbi.nlm.nih.gov/pubmed/?term=Fair%20JM%5BAuthor%5D&cauthor=true&cauthor_uid=20231759), [Haskell WL](http://www.ncbi.nlm.nih.gov/pubmed/?term=Haskell%20WL%5BAuthor%5D&cauthor=true&cauthor_uid=20231759), et al. Validation of the Stanford Brief Activity Survey: examining psychological factors and physical activity levels in older adults. [J Phys Act Health](http://www.ncbi.nlm.nih.gov/pubmed/?term=Validation+of+the+Stanford+Brief+Activity+Survey%3A+Examining+Psychological+Factors+and+Physical+Activity+Levels+in+Older+Adults) 2010;7:87-94.
3. World Health Organization: Global Strategy on Diet, Physical Activity and Health. In Proceedings of the 57th World Health Assembly. Geneva, Switzerland: World Health Organization; 2004:2–18.
4. Singh A, Purohit B: Evaluation of Global Physical activity Questionnaire (GPAQ) among healthy and obese health professionals in central India. Baltic J Health Phys Act 2011, 3:34–43.
5. Bull FC, Maslin TS, Armstrong T. Global physical activity questionnaire (GPAQ) nine country reliability and validity study. J Phys Act Health 2009:6:790–804.
6. Herrmann SD, Heumann KJ, Der Ananian CA, Ainsworth BE. Validity and reliability of the Global Physical Activity Questionnaire (GPAQ). Meas Phys Educ Exerc Sci 2013:17:221–235.
7. [Cleland CL](http://www.ncbi.nlm.nih.gov/pubmed/?term=Cleland%20CL%5BAuthor%5D&cauthor=true&cauthor_uid=25492375), [Hunter RF](http://www.ncbi.nlm.nih.gov/pubmed/?term=Hunter%20RF%5BAuthor%5D&cauthor=true&cauthor_uid=25492375), [Kee F](http://www.ncbi.nlm.nih.gov/pubmed/?term=Kee%20F%5BAuthor%5D&cauthor=true&cauthor_uid=25492375), [Cupples ME](http://www.ncbi.nlm.nih.gov/pubmed/?term=Cupples%20ME%5BAuthor%5D&cauthor=true&cauthor_uid=25492375), [Sallis JF](http://www.ncbi.nlm.nih.gov/pubmed/?term=Sallis%20JF%5BAuthor%5D&cauthor=true&cauthor_uid=25492375), [Tully MA](http://www.ncbi.nlm.nih.gov/pubmed/?term=Tully%20MA%5BAuthor%5D&cauthor=true&cauthor_uid=25492375). Validity of the global physical activity questionnaire (GPAQ) in assessing levels and change in moderate-vigorous physical activity and sedentary behaviour. [BMC Public Health](http://www.ncbi.nlm.nih.gov/pubmed/25492375) 2014;14:1255.
8. Au TB, Blizzard L, Schmidt M, Pham LH, Magnussen C, Dwyer T. Reliability and validity of the global physical activity questionnaire in Vietnam. J Phys Act Health 2010:7:410–418.
9. Hoos T, Espinoza N, Marshall S, Arredondo EM. Validity of the Global Physical Activity Questionnaire (GPAQ) in adult Latinas. J Phys Act Health 2012, 9:698–705.
